# Using Bayesian modelling to investigate factors governing antibiotic-induced *Candida albicans* colonization of the GI tract

**DOI:** 10.1038/srep08131

**Published:** 2015-02-03

**Authors:** Jyoti Shankar, Norma V. Solis, Stephanie Mounaud, Sebastian Szpakowski, Hong Liu, Liliana Losada, William C. Nierman, Scott G. Filler

**Affiliations:** 1 J. Craig Venter Institute, Rockville, MD, USA; 2 Los Angeles Biomedical Research Institute at Harbor-UCLA Medical Center, Torrance, CA, USA; 3 David Geffen School of Medicine at UCLA, Los Angeles, CA, USA

## Abstract

Receipt of broad-spectrum antibiotics enhances *Candida albicans* colonization of the GI tract, a risk factor for haematogenously-disseminated candidiasis. To understand how antibiotics influence *C. albicans* colonization, we treated mice orally with vancomycin or a combination of penicillin, streptomycin, and gentamicin (PSG) and then inoculated them with *C. albicans* by gavage. Only PSG treatment resulted in sustained, high-level GI colonization with *C. albicans*. Furthermore, PSG reduced bacterial diversity in the colon much more than vancomycin. Both antibiotic regimens significantly reduced IL-17A, IL-21, IL-22 and IFN-γ mRNA levels in the terminal ileum but had limited effect on the GI fungal microbiome. Through a series of models that employed Bayesian model averaging, we investigated the associations between antibiotic treatment, GI microbiota, and host immune response and their collective impact on *C. albicans* colonization. Our analysis revealed that bacterial genera were typically associated with either *C. albicans* colonization or altered cytokine expression but not with both. The only exception was *Veillonella*, which was associated with both increased *C. albicans* colonization and reduced IL-21 expression. Overall, antibiotic-induced changes in the bacterial microbiome were much more consistent determinants of *C. albicans* colonization than either the GI fungal microbiota or the GI immune response.

The fungus *Candida albicans* is a human commensal that grows on the skin and mucosal surfaces of healthy individuals^1^. However, in susceptible patients, *C. albicans* can enter the bloodstream, either by translocation across the mucosa of the gastrointestinal (GI) tract^2^ or via an indwelling vascular catheter^3^. The resulting haematogenously-disseminated infection is associated with a mortality of up to 49% and is the fourth most common cause of hospital-acquired bloodstream infections in the U.S.^3^.

Risk factors for developing disseminated and invasive candidiasis include the receipt of broad-spectrum antibiotics, immunosuppression, and colonization with *Candida* species. It is believed that broad-spectrum antibiotics predispose patients to developing disseminated candidiasis by suppressing the competing bacterial microbiota in the GI tract and encouraging the overgrowth of *C. albicans*^2^,^4^,^5^,^6^,^7^. In recent randomized clinical trials, probiotics were effective in reducing GI colonization by *Candida* species and decreasing the incidence of invasive candidiasis^8^,^9^. In immunodeficient and malnourished mice, introduction of *Lactobacillus* species into the GI tract decreased GI *C. albicans* burden and resulted in lower mortality^10^,^11^. These results suggest that the composition of the GI tract microbiota influences the level of *C. albicans* colonization.

Another potential influence on the level of *C. albicans* GI colonization is the host immune status^12^. Mutations in genes that code for host cytokines such as interleukin (IL) -17, IL-22 and interferon (IFN) -γ increase susceptibility to mucosal candidiasis in both mice and humans^12^,^13^. T-cell effector populations that drive cytokine responses express receptors for peptide antigens of specific commensal bacteria^14^. Among these, gram-positive bacilli from the genus *Clostridium*^15^ and the closely related genus, “*Candidatus Arthromitus*” also known as the segmented filamentous bacteria (SFB), are prominent activators of adaptive Th17 immunity in the GI tract^16^,^17^,^18^. By depleting these immune-activating bacteria^2^ and through possible direct immunomodulation^19^, antibiotics have the potential to enhance *C. albicans* GI colonization.

A mechanistic understanding of the tri-faceted interface of antibiotic action on the local immune response, GI bacterial and fungal microbiota, and *C. albicans* colonization is essential for developing strategies to reduce the incidence of candidiasis. Antibiotic regimens that include penicillin are associated with substantially higher rates of GI colonization with *Candida* species when compared with those without penicillin^20^,^21^. Therefore, to examine the factors that influence the level of *C. albicans* GI colonization, we contrasted the effects of a combination of penicillin, streptomycin and gentamicin (PSG) with those of vancomycin in a mouse model. We studied two GI sites: a) the terminal ileum because the small intestine contains a large number of immune cells, particularly those involved in a Th17 response^16^, and b) the colon (sampled via faecal pellets), which is the most commonly sampled site for profiling the microbiome^22^,^23^. Unlike humans, mice are not normally colonized with *C. albicans* unless the resident GI flora is perturbed by the administration of oral antibiotics^21^. Hence, the mouse model provided us a controlled platform to perform our experiments.

Mouse studies that incorporate next generation sequencing (NGS) for measuring microbiome variables represent a high-dimensional setting where the number of measurements substantially exceeds the number of samples. As an example, an experiment with 30 mice can generate over 300 bacterial, fungal and immune measurements per mouse. As a consequence of this high-dimensionality, a large number of equally likely models with various combinations of microbes and other covariables can explain antibiotic effects and subsequent colonization^24^. However, most current microbiome studies do not systematically explore this large model space. Instead, a number of these studies apply a univariate method such as the one-way ANOVA test, Wilcoxon rank-sum test or Kruskal-Wallis rank-sum test to individually screen each measured microbiome variable for a significant effect. This significance testing typically includes correction strategies for multiple testing. For any response of interest, the effects of the significant microbes selected by the univariate method are then estimated using a single regression model^25^.

While univariate microbiome variable selection followed by a single regression model has yielded important new information about factors that affect the composition of microbiomes, the p-values or confidence intervals (CI) that are computed after univariate screening often overestimate the strength of conclusions^26^,^27^. Even with corrections for multiple testing in place, independent tests do not consider the complex multivariable, correlational structure of microbiome data. Furthermore, the final step of inference is based upon a single model that ignores the uncertainty that compounds with each variable selection step prior to building the final model^28^. Ensemble multivariable regression modelling approaches such as Bayesian model averaging (BMA) have been used in ecology to address these shortcomings^29^. Ensemble methods examine a large number of variable configurations to identify significant variables and to simultaneously compute their effect sizes. After a systematic evaluation of several such ensemble approaches on our mouse microbiome data, we selected BMA for its strong performance across several variable selection and ranking metrics^30^. In addition, BMA helped us formally account for model uncertainty in high-dimensions by simultaneously computing two crucial statistical metrics for each finding: a) consistency as measured by posterior inclusion probability (PIP), and b) statistical significance using the 95% CI of effect size^31^. Thus, BMA has a distinct advantage over most statistical approaches, which typically provide estimates of statistical significance but not of consistency.

We employed BMA to identify the microbiome community and the host immune response signatures that characterize antibiotic treatment, and to probabilistically rank the influence of the microbiome and the host immune response on the level of *C. albicans* colonization of the mouse GI tract following gastric challenge with this organism. Our analysis revealed that antibiotic-induced changes in the bacterial microbiome were the most consistent determinants of *C. albicans* colonization. These bacterial genera typically influenced the growth of *C. albicans* without simultaneously altering the host GI immune response. Although antibiotics significantly altered the composition of GI fungal microbiota and suppressed the GI immune response, these factors were less influential on colonization relative to the bacterial microbiota.

## Results

### PSG and vancomycin had differential effects on *C. albicans* colonization and microbial diversity, but induced similar host immune responses

**Figure 1:**
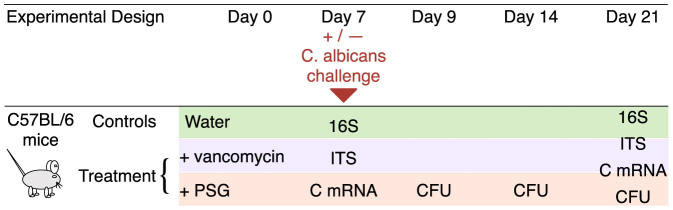
Experimental Design. Following initiation of antibiotics, and inoculation with *C. albicans*, we collected samples of the mouse terminal ileum and faecal pellets. We then sequenced the bacterial 16S and fungal ITS amplicons in the samples by 454 sequencing and taxonomically classified these sequences using a tailored bioinformatics workflow. We quantified mRNA expression levels of 6 host cytokines (C mRNA) including IL-17A, IL-21, IL-22, IFN-γ, TNF-α and IL-4 from segments of the terminal ileum. Using quantitative culture, we determined the level of *C. albicans* colonization in the faecal pellets in terms of colony forming units per gram (CFU/g).

We used a mouse model to investigate the tri-faceted effect of antibiotics on the host immune response, the GI bacterial and fungal microbiota, and *C. albicans* colonization in the GI tract. Mice in the control group received sterile drinking water, while those in treatment groups received sterile drinking water containing either vancomycin or PSG. After 7 days, we inoculated some of the mice in the control and treatment groups with 10^5^ *C. albicans* cells, administered by gavage (Figure 1). We then continued the mice on their original control or treatment regimens for an additional 14 days. Each group had 3 to 7 mice, and the results of at least two independent experiments were combined (details in Methods).

**Figure 2:**
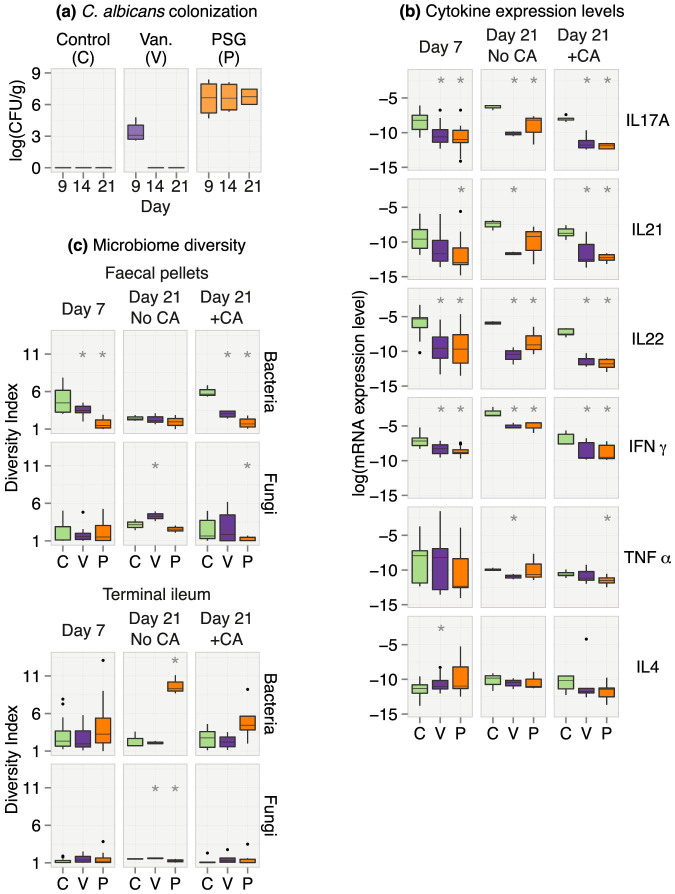
Exploring the effects of PSG (P) and vancomycin (Van., V) relative to controls on: (a) *C. albicans* colonization: Levels of log transformed colony forming units per gram (CFU/g) of *C. albicans* from faecal pellets sampled on days 9, 14 and 21 from controls (green), vancomycin-treated (violet) and PSG-treated (orange) mice. (b) Cytokine expression levels: mRNA expression levels of the 6 cytokines measured from segments of the terminal ileum sampled on days 7 and 21. +CA and No CA respectively indicate mice groups with and without exposure to *C. albicans* gavage. (c) Microbiome diversity: bacterial and fungal diversity on days 7 and 21 both with (+CA) and without *C. albicans* exposure (No CA) in the faecal pellets and the terminal ileum. Diversity was computed using the inverse Simpson index. Statistical analysis: In each boxplot, the upper and lower hinges correspond to the 25th and 75th percentiles of the data, also known as the interquartile range (IQR). The upper and lower whiskers extend from the hinge to the highest value within 1.5 times the IQR. Points beyond the end of the whiskers denote outliers. Asterisks, when present, denote statistically significant differences relative to controls. Findings were considered statistically significant when the 99.6% bootstrap confidence intervals (CI) of the normalized mean difference (Welch's statistic) between the antibiotic group and the controls did not include zero. The 99.6% bootstrap CIs were computed over 10,000 resamples and included a conservative correction for 6 simultaneous hypotheses corresponding to the 6 cytokines. Results shown are the combined data from at least two independent experiments of 3–5 mice each.

Following exposure to *C. albicans*, the faecal pellets of control mice contained no detectable *C. albicans* cells by quantitative culture on days 9, 14 and 21 (Figure 2a). In contrast, mice that received PSG had sustained, high-level of colonization with *C. albicans* that persisted throughout the duration of the experiment. Unlike PSG treatment, vancomycin treatment induced only a low-level, transient colonization on day 9, and no *C. albicans* cells were cultured from the faecal pellets on days 14 and 21.

Next, we determined the local GI immune response in the various treatment groups using real-time PCR to measure the mRNA expression levels of six cytokines in the terminal ileum. These cytokines consisted of IL-4, IL-17A, IL-21, IL-22, IFN-γ, and tumour necrosis factor (TNF) -α. We selected these cytokines because they have been shown to be relevant in the context of *C. albicans* colonization of the GI tract^32^. Relative to the controls, treatment with either vancomycin or PSG was associated with significantly depressed expression levels of IL-17A, IL-22 and IFN-γ on both days 7 and 21, both in the presence and absence of *C. albicans* exposure (Figure 2b).

Finally, we compared the bacterial and fungal diversity, using the inverse Simpson diversity index, in PSG and vancomycin-treated mice relative to controls. The GI bacterial and fungal microbiomes were profiled by sequencing the bacterial 16S rRNA and fungal ITS regions followed by taxonomic classification. Across our experiments, we detected 344 bacterial genera and 109 fungal genera. In the faecal pellets, both vancomycin and PSG treatments significantly decreased bacterial diversity on day 7 and day 21 in the presence of *C. albicans*, relative to controls. PSG treatment caused a greater reduction in diversity, as expected from the broader spectrum of activity of this antibiotic combination (Figure 2c). In the terminal ileum, treatment with vancomycin did not affect bacterial diversity, while treatment with PSG resulted in an increase in diversity, more so in the absence of *C. albicans* exposure (Figure 2c).

Compared to bacterial diversity, fungal diversity was lower in both the colon and terminal ileum. Two weeks after *C. albicans* challenge, there was an increase in fungal diversity in the vancomycin-treated mice in the faecal pellets. In contrast, the fungal diversity remained low in the PSG-treated mice (Figure 2c). In comparison to the faecal pellets, the fungal diversity in the terminal ileum of the controls was lower. Collectively, these results indicate that vancomycin and PSG had different effects on microbial diversity in the GI tract. Moreover, the effect of the antibiotics differed across locations in the GI tract.

Thus our exploratory analysis of the three facets of the microbiome-immune-colonization interface revealed that both vancomycin and PSG reduced bacterial diversity in the faecal pellets and suppressed cytokine mRNA expression in the terminal ileum. However, only PSG induced susceptibility to sustained *C. albicans* colonization.

### The antibiotic-microbiome interface: PSG and vancomycin resulted in markedly different bacterial and fungal microbiome signatures

**Figure 3:**
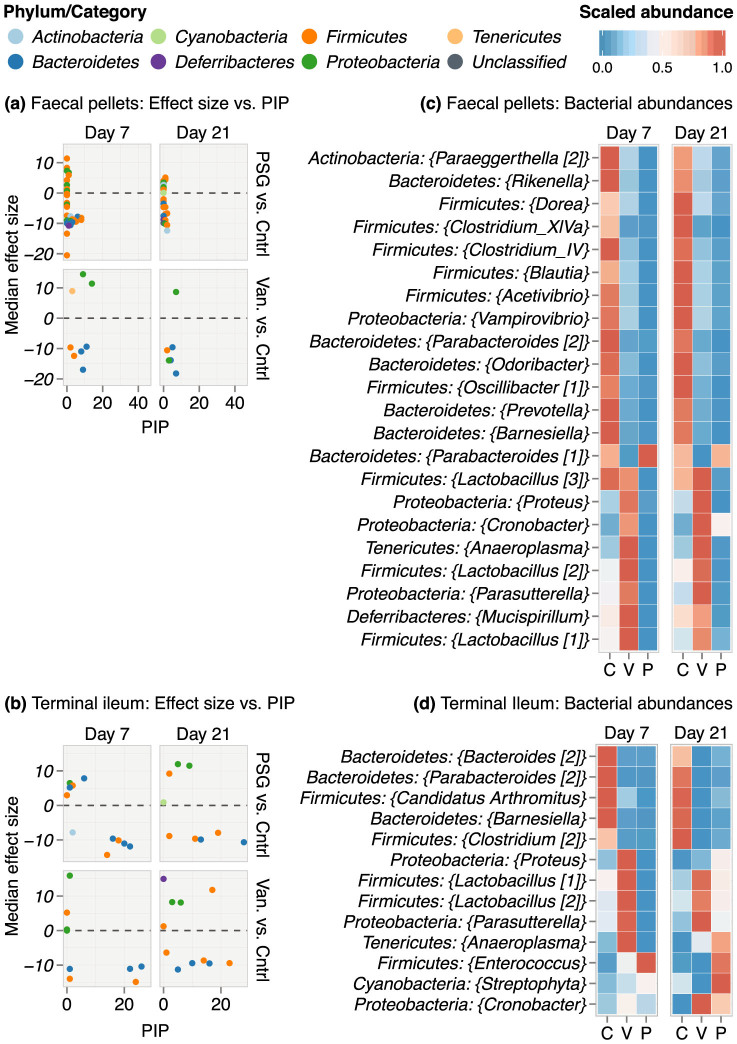
Effect of antibiotics on the bacterial microbiome. Results from BMA logistic regression ensembles exploring the antibiotic and bacterial microbiome interface are shown in panels (a) and (c) for the faecal pellets and (b) and (d) for the terminal ileum. Panels (a) and (b) plot the median effect size of each bacterium against its posterior inclusion probability (PIP) in the model. PIP is expressed in %. The effect size of a microbe is its regression coefficient in the model and depends on its relative abundance under antibiotic treatment (PSG or Van.) relative to controls (Cntrl), after adjusting for all the other covariables in the model. We show only the effects that were statistically significant i.e. the corresponding Bayesian 95% CIs did not include zero. Higher PIPs indicate higher consistency in antibiotic effects. PIPs can range from 0% (not consistent) to 100% (very highly consistent). All models were built at the genus level. Each point in the graph denotes a genus coloured by its phylum membership. Panels (c) and (d) show heatmaps of relative abundances (scaled to the range 0–1) of bacteria with the top consistent and significant differences across the PSG (P), vancomycin (Van., V) and control (C) groups on days 7 and 21. Each genus is annotated with its phylum-level label in the heatmaps. Numbers in square brackets denote distinct sequence clusters that were mapped to the same genus but could not be collapsed since they were more than 3% dissimilar from each other.

We next identified the set of specific bacterial and fungal genera that were significantly and consistently altered by either PSG or vancomycin relative to controls by building multivariable BMA logistic regression models (Figure 3)^33^. BMA explored a set of 10,000 genera configurations that differentiated PSG and vancomycin from controls. The posterior inclusion probability (PIP) for any genus under BMA is the relative frequency with which it was selected as influential across these 10,000 configurations^31^. Genera with higher PIPs were able to consistently differentiate antibiotics versus control status across samples.

The effect size for a given genus is its regression coefficient in the model and is determined by the difference in its relative abundance between antibiotic treated mice and control mice, after adjusting for all the other microbe proportions and cytokine variables in the model. We built separate models to examine the effects of antibiotics on the bacterial and fungal microbiota in the faecal pellets and in the terminal ileum for days 7 and 21 (Figure 3). We show only the genera whose 95% CI of effect sizes did not include zero and thus, represent statistically significant antibiotic effects.

Overall, the maximum PIPs in the antibiotic-microbiome models were approximately 30%, indicating that neither PSG nor vancomycin had a consistent impact on any given genus across samples (Figures 3a and b). In the faecal pellets, the effects of PSG on the bacterial microbiome were the most heterogeneous, with a large number of high and significant effect sizes accompanied by relatively low PIPs (Figure 3a). Unlike PSG, vancomycin was associated with a lower number of significant effects. However, these effects were more consistent. In the terminal ileum, PSG and vancomycin had a similar pattern of effects on the bacterial microbiome, and the signatures in the ileum were more consistent than those in the faecal pellets (Figure 3b). In both GI sites, bacterial genera belonging to the *Bacteroidetes* and *Firmicutes* phyla had the most consistent antibiotic signatures with higher PIPs.

In the faecal pellets, PSG consistently and significantly suppressed most genera on both days 7 and 21 (Figure 3c). Most of these genera were members of phyla *Bacteroidetes* and *Firmicutes*. By contrast, *Parabacteroides* was the only genus that increased in relative abundance in the PSG-treated mice on days 7 and 21. Even though vancomycin reduced the relative abundance of several of the same genera as did PSG, the magnitude of the reduction was smaller in comparison. In addition, vancomycin increased multiple genera, including *Lactobacillus*, *Proteus*, *Cronobacter*, *Anaeroplasma*, *Parasutterella* and *Mucispirillum*.

In the terminal ileum, PSG treatment had a mixed effect, decreasing the relative abundance of some genera, but increasing others (Figure 3d). The increase in *Enterococcus*, *Streptophyta* and *Anaeroplasma* was especially notable at day 21 in the presence of *C. albicans* colonization. Vancomycin also had a similar mixed effect. It depleted the same genera as did PSG, including “*Candidatus Arthromitus*” which is known to stimulate a Th17 response in the GI tract^15^,^16^. However, vancomycin treatment increased a greater number of genera than did PSG, especially on day 7.

**Figure 4:**
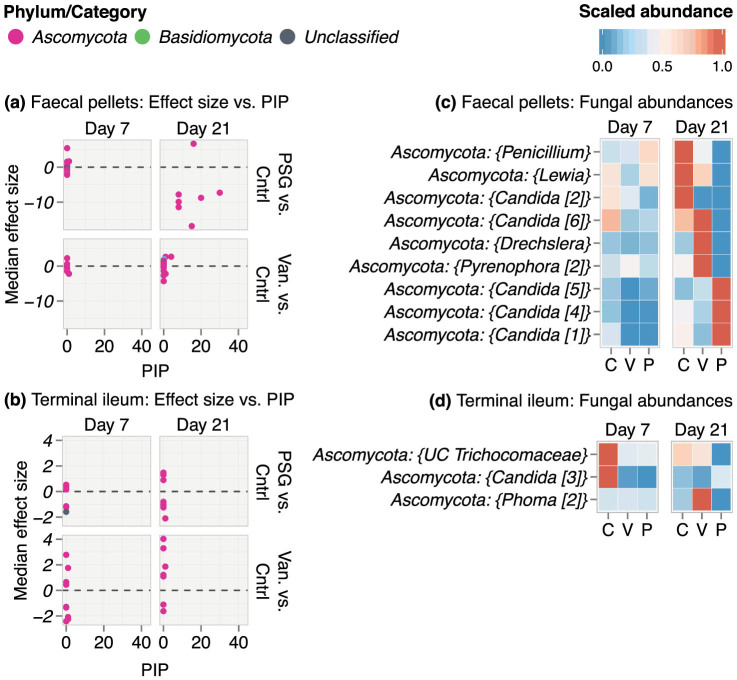
Effect of antibiotics on the fungal microbiome. Results from BMA logistic regression ensembles exploring the antibiotic and fungal microbiome interface are shown in panels (a) and (c) for the faecal pellets and (b) and (d) for the terminal ileum. Panels (a) and (b) plot the median effect size of each fungus against its posterior inclusion probability (PIP) in the model. PIP is expressed in %. The effect size of a microbe is its regression coefficient in the model and depends on its relative abundance under antibiotic treatment (PSG or Van.) relative to controls (Cntrl), after adjusting for all the other covariables in the model. We show only the effects that were statistically significant i.e. the corresponding Bayesian 95% CIs did not include zero. Higher PIPs indicate higher consistency in antibiotic effects. PIPs can range from 0% (not consistent) to 100% (very highly consistent). All models were built at the genus level. Each point in the graph denotes a genus coloured by its phylum membership. Panels (c) and (d) show heatmaps of abundances (scaled to the range 0–1) of fungi with the top consistent and significant differences across the PSG (P), vancomycin (Van., V) and control (C) groups on days 7 and 21. Each genus is annotated with its phylum-level label in the heatmaps. Numbers in square brackets denote distinct sequence clusters that were mapped to the same genus but could not be collapsed since they were more than 3% dissimilar from each other. UC denotes sequence clusters that did not have a genus level classification in any of the databases (NCBI, SILVA) we used as reference for taxonomic classification and could not be resolved using the Megablast algorithm.

On day 7, the fungal microbiome of mice receiving either PSG or vancomycin had substantial mouse-to-mouse variability in both GI sites (Figure 4a and 4b). However, by day 21, PSG induced a larger number of consistent effects in the fungal genera of the faecal pellets than did vancomycin. Five of these effects were on sequence clusters that mapped to *Candida* (Figure 4c). Of these, *Candida* [2] decreased under both antibiotics while *Candida* [1], [4] and [5] increased only under PSG. *Candida* [6] was present in a higher proportion in controls, increased under vancomycin and decreased under PSG.

In the terminal ileum, neither antibiotic had consistent effects on the fungal microbiome.

We were specifically interested in obtaining insights into *Candida* colonization and therefore explored the species level composition of *Candida* clusters that were identified as influential by the BMA approach. Using Megablast^34^, we found that the sequence clusters labelled Candida [1], [2], and [4] were predominantly composed of *C. albicans*, *Candida* [6] was primarily *Candida tropicalis*, while *Candida* [5] had a mixed composition. These results suggest that *C. albicans* likely displaced the other fungi in the faecal pellets, including other *Candida* species such as *C. tropicalis* that were present in the mouse gut before colonization. It was also notable that *C. tropicalis* was detectable only by ITS sequencing and not by quantitative culture suggesting a possible specific adaptation to the conditions of the GI tract and an inability to grow on the standard fungal medium that was used.

### The antibiotic-microbiome-cytokine interface: Antibiotic treatment influenced cytokine mRNA levels primarily through effects on the bacterial and not the fungal microbiota

Our exploratory analysis showed that both antibiotics significantly suppressed cytokine mRNA levels in the terminal ileum. We built BMA linear regression models to identify the underlying changes in microbial genera associated with the altered cytokine expression. In these models, the cytokine mRNA expression level served as the continuous response. The microbiota and categorical constructs representing antibiotic treatment, and *C. albicans* exposure served as the independent covariables. We estimated a separate model for each cytokine.

**Figure 5:**
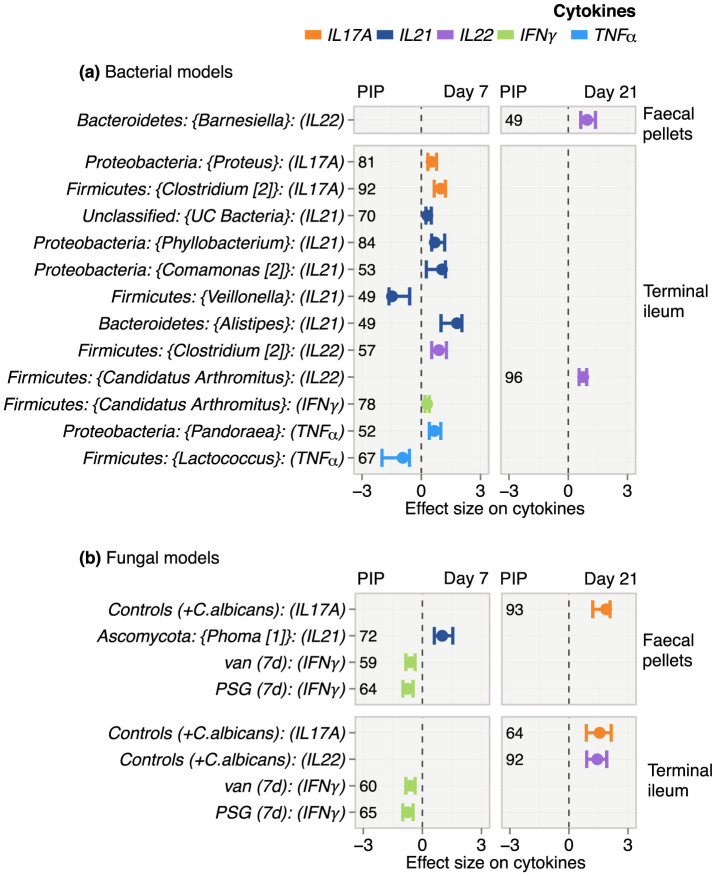
Effect of the microbiome and antibiotic treatment on host cytokine mRNA expression. Results from BMA linear regression ensembles that explain cytokine expression level as a function of the microbiome and antibiotic treatment. A separate model was estimated for each cytokine. Panels (a) and (b) show findings in the bacterial and fungal models, respectively. We show only the effects that were statistically significant i.e. the corresponding Bayesian 95% CIs did not include zero. The effect sizes and the Bayesian 95% CIs of the top consistent and significant variables are presented. A higher value of PIP (expressed in %) indicates that the variable is consistently associated with cytokine expression levels across the space of models explored in BMA.

In the faecal pellets, the only genus with a fairly consistent positive effect on any cytokine was *Barnesiella*, which was positively associated with IL-22 at day 21 (PIP = 49%) (Figure 5a). In contrast, we observed several consistent effects in the terminal ileum especially on day 7. The genus *Clostridium* and the related genus, “*Candidatus Arthromitus*” had the most consistent and significant influence on cytokines. On day 7, Clostridium [2] was positively associated with IL-17A (PIP = 92%) and IL-22 (PIP = 57%) while “*Candidatus Arthromitus*” was positively associated with IFN-γ (PIP = 78%). Other consistent positive associations in the terminal ileum involved genera of the phylum *Proteobacteria*, including *Phyllobacterium* on IL-21 (PIP = 84%), *Proteus* on IL-17A (PIP = 81%) and *Comamonas* on IL-21 (PIP = 53%). The only genera with negative cytokine associations were *Lactococcus* on TNF-α (PIP = 67%) and *Veillonella* on IL-21 (PIP = 49%). On day 21, the only highly consistent positive effect was that of “*Candidatus Arthromitus*” on IL-22 (PIP = 96%). In the fungal models (Figure 5b), the only genus with a consistently positive association with IL-21 on day 7 was *Phoma* in the faecal pellets (PIP = 72%). On day 7, treatment with either PSG or vancomycin had a negative influence on IFN-γ in both GI sites. On day 21, the introduction of *C. albicans* without concurrent antibiotic treatment was associated with increased IL-17A mRNA levels in both GI sites, and with increased IL-22 in the faecal pellets.

Overall, both bacterial and fungal genera had relatively consistent positive associations with the panel of cytokines that were studied. Most of these bacteria were depleted due to antibiotic administration, thus explaining the decrease in the overall cytokine levels that we observed in our exploratory analyses (Figure 3b). The negative association of antibiotic treatment with IFN-γ only surfaced in the fungal model on day 7. Similarly, the positive effects of *C. albicans* on IL-17A and IL-22 only appeared in the fungal model. These differences between the bacterial and fungal models likely represent the stronger effects of the bacteria on the cytokines as compared to the fungi. The strong antibiotic effects on cytokines within the fungal models indicate that the antibiotic variables were proxies for the relevant effects of antibiotics on the bacterial microbiome.

### The antibiotic-microbiome-cytokine-colonization interface: Antibiotic-shaped bacterial genera explained levels of *C. albicans* colonization more consistently than either fungal genera or cytokines

**Figure 6:**
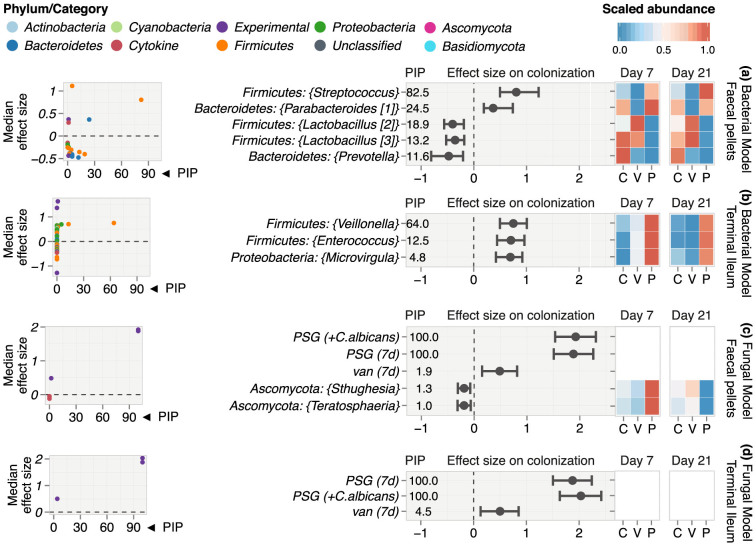
Effect of the microbiome, cytokine expression and antibiotic treatment on *C. albicans* colonization. Results from BMA linear regression ensembles that explain colonization in log(CFU/g) as a function of the microbiome, cytokine expression levels and antibiotic treatment. Findings from the bacterial models are presented in panels (a) (faecal pellets) and (b) (terminal ileum) while those from the fungal models are presented in panels (c) (faecal pellets) and (d) (terminal ileum). From left to right, each panel consists of (left) a plot of effect size vs. PIP, (middle) 95% Bayesian CI of effect sizes and (right) heatmaps of scaled microbe abundances across control and treatment groups on days 7 and 21. All analyses were performed at the genus level. In the plots showing effect size vs. PIP, each point denotes a variable coloured by its phylum membership or category (e.g. “Experimental”). PIP is expressed in %. A higher value of PIP indicates that the variable is consistently associated with colonization across the space of models explored in BMA. All plots show only the top consistent variables that attained the highest PIPs and whose effects on *C. albicans* colonization were statistically significant i.e. the corresponding Bayesian 95% CIs did not include zero. The heatmaps show relative abundances (scaled to the range 0–1) of the most influential bacteria and fungi that consistently explained levels of *C. albicans* colonization across PSG (P), vancomycin (V) and controls (C) groups on days 7 and 21.

In our final set of multivariable BMA models, we identified the microbiota and cytokines most influential on the level of *C. albicans* colonization. We built separate BMA linear regression models for bacterial and fungal genera and one for each GI site (Figure 6). The covariables in the models consisted of the microbial genera, cytokine mRNA expression levels, day of sampling, antibiotic treatment, and *C. albicans* exposure. The level of *C. albicans* colonization (measured as CFUs in the faecal pellets) was the continuous response variable.

In each of the models, only a handful of variables obtained a high PIP (Figure 6). Genera from the phylum *Firmicutes* had the highest PIP in both the faecal pellets and the terminal ileum (Figure 6a and b). In the faecal pellets, the bacterial genera with the highest PIPs were *Streptococcus* and *Parabacteroides* (Figure 6a). Both were positively associated with higher levels of *C. albicans* colonization and had increased relative abundance in PSG-treated mice. *Lactobacillus* and *Prevotella* were protective against colonization, although they had lower PIPs. While the relative abundance of *Prevotella* was higher in the control mice, the relative abundance of *Lactobacillus* was higher mainly in the vancomycin-treated mice. In the terminal ileum, *Veillonella* and to a lesser extent, *Enterococcus*, were the primary genera positively associated with higher levels of colonization (Figure 6b). Both these genera increased under PSG treatment.

In the fungal models, variables encoding PSG treatment both with and without concomitant *C. albicans* exposure were the most influential, with a large positive effect on colonization and a PIP of 100% (Figure 6c and d). All the other fungal and cytokine variables had very low PIPs. When this finding is viewed together with the high PIPs assigned to specific bacterial genera in the bacterial model, it is likely that the exclusion of these bacteria in the fungal model resulted in high PIP assignment to the categorical variables for PSG treatment and *C. albicans* exposure, which served as proxies for the bacterial effects.

Using model diagnostics (see supplementary information for details), we determined that the bacterial model estimated from the faecal pellets was able to explain as much as 90% of the observed variation in *C. albicans* colonization levels, thus demonstrating good explanatory power. Furthermore, the intercept in the bacterial model had a very low PIP (≈0%) indicating that the model was estimated using a large fraction of the relevant variables influential on *C. albicans* colonization levels.

The combined findings from the bacterial and fungal models indicate that antibiotic-induced changes in the GI bacterial microbiota and the antibiotics themselves constituted a far more influential effect on colonization than either the resident GI fungi or cytokines.

## Discussion

Broad-spectrum antibiotics are known to have a wide-ranging impact on the gut microbiome^35^ and immunomodulatory effects on both the innate and adaptive components of the immune system^19^,^36^. Both the GI flora and the local GI immune response have the potential to prevent or limit GI colonization by *C. albicans*. In this study, we employed BMA, a Bayesian modelling approach, to examine the complex interface connecting antibiotics, the microbiome, cytokines and *C. albicans* colonization. Within each facet of this interface, BMA enabled us to identify the most influential variables and rank their relative contributions. Our results indicate that members of the antibiotic-influenced bacterial microbiome had the most consistent and substantial influence on *C. albicans* colonization than either the fungal microbiome or the local immune response.

Other groups have studied experimental setups that are similar to ours^4^,^5^,^6^,^7^. However, our experiments differ from these studies in several important aspects. Our primary focus was on obtaining mechanistic insights into *C. albicans* colonization during a longer span of antibiotic treatment (21 days). In contrast, the earlier studies examined either reassembly of the microbiome after discontinuation of a short duration of antibiotics in the presence and absence of *C. albicans* exposure^4^,^5^,^6^, or the impact of very long-term antibiotics without a specific focus on *C. albicans* colonization^7^. While these studies primarily examined the differential abundance of microbiota across treatments, our modelling framework extends these analyses to simultaneously examine the impact of long-term antibiotics on the gut microbiome and the host immune response and their combined influence on *C. albicans* colonization. Furthermore, we studied colonization as a quantitative response by contrasting the effects of two antibiotic treatments (vancomycin and PSG) that induce differential levels of *C. albicans* colonization.

Mason et al.^5^ showed that introducing *C. albicans* prevents *Lactobacillus* species from repopulating the GI tract post-antibiotics and promotes the growth of *Enterococcus faecalis*. We complement their findings by showing that *Lactobacillus* is associated with protection against *C. albicans* colonization in the faecal pellets, while *Enterococcus* in the terminal ileum is positively associated with *C. albicans* colonization. We also found that treatment with vancomycin was associated with enhanced growth of *Lactobacillus* in the faecal pellets. It is thus tempting to speculate that the increased growth of this inhibitory genus was one of the reasons why mice that received vancomycin had only transient *C. albicans* colonization.

We further extend these mechanistic insights by showing that in the faecal pellets, *Streptococcus* and *Parabacteroides* appeared to promote *C. albicans* colonization and thus act antagonistically to *Lactobacillus* and *Prevotella*. *Streptococcus* was unique because this genus was consistently associated with colonization but not with either antibiotics or cytokines. This finding suggests that the colonization promoting action of *Streptococcus* involves pathways that do not involve either the immune response panel that we examined or the differential abundance effects induced by the antibiotics. This ties in well with recent research that suggests that *Streptococci* and *Candida* species interact through several molecular mechanisms to promote synergistic infection of the oral mucosa^37^,^38^. It is possible that similar mechanisms are prevalent in the GI tract.

In the cytokine models, we identified consistent positive effects of the genus *Clostridium* on the expression of IL-17A and IL-22 mRNA. The closely related genus “*Candidatus Arthromitus*” was also found to strongly promote IL-22 and IFN-γ, and was the only genus to strongly influence the cytokine response on day 21. These results are in agreement with previous reports of the stimulatory effects of “*Candidatus Arthromitus*” on the GI immune response^8^,^16^,^17^,^18^,^39^,^40^. Furthermore, we identified several other genera, such as *Phyllobacterium*, *Proteus*, *Comamonas*, *Lactococcus* and *Pandoraea* that likely influence GI cytokine mRNA levels in the terminal ileum. While these results need to be verified experimentally, they demonstrate the power of BMA to identify bacteria that may play important roles in shaping the GI immune response.

It was notable that neither *Clostridia* nor “*Candidatus Arthromitus*” were associated with *C. albicans* colonization. Conversely, other bacteria such as *Streptococcus* in the faecal pellets were strongly associated with *C. albicans* colonization but not with cytokine mRNA levels. These contrasting results suggest that in the faecal pellets, the bacterial influence on *C. albicans* colonization was either direct or mediated by other mechanisms that did not involve the host GI immune response. A possible exception is *Veillonella*, which was associated with both suppression of IL-21 and stimulation of *C. albicans* colonization in the terminal ileum. Our findings are supported by recent work by Vautier et al.^41^ who showed that both IL-17 knockout mice and mice treated with an IL-1 blocker had normal levels of *C. albicans* GI colonization, which suggests that these cytokines may not play a significant role in controlling the level of colonization. Given these interesting and varied associations between the microbiota and host immune response, we expect that analysing an expanded panel of cytokines and immune effector cells could yield further insights into the host factors that govern *C. albicans* colonization.

Fungi other than *C. albicans* appeared to be largely displaced by *C. albicans* colonization and, in addition, were not a substantial influence on the host cytokine expression. Among these displaced fungi were those that mapped to *C. tropicalis*. However, we could not detect this species using quantitative culture. Our findings are thus in agreement with those reported by Iliev et al.^42^ and suggest that the strain(s) of *C. tropicalis* that grow in in the mouse GI tract may not grow under standard culture conditions.

A limitation of the current work is that our models did not incorporate absolute microbiota counts. However, model diagnostics showed that the BMA ensembles were able to explain up to 90% of the observed variation in *C. albicans* colonization levels. This indicates that changes in the absolute microbiota counts would be unlikely to add substantially to the model explaining *C. albicans* colonization levels, over and above the explanation afforded by the variables already in the model, including relative abundances of microbiota, cytokine expression levels and other experimental variables. Nevertheless, it is possible that absolute microbiota counts may constitute a more direct influence on colonization by other microorganisms in alternative models.

It is possible to apply frequentist stability-based approaches to explore large model spaces for variable selection in microbiome analysis^43^. However, the key advantage of BMA is its ability to explore a much larger number of microbiome and cytokine variable configurations. Unlike frequentist approaches, BMA can simultaneously identify the most influential variables, capture heterogeneity in the effects of these variables, and provide uncertainty estimates around the effect sizes without any assumptions about their distributions^31^,^44^. Employing BMA substantially reduces the likelihood of false positives that typically accumulate in multi-step modelling approaches.

BMA findings suggest that there were many substantial effects of individual members of the microbiome community on *C. albicans* colonization that were not uniformly strong across the samples. Although a majority of these effects may not be important by themselves, the combination of several such weak effects across the microbiome community likely constituted a substantial influence on *C. albicans* colonization.

Thus, BMA provided a broader insight into the nature and distribution of effects within the data. Incorporating versatile statistical methods such as BMA in microbiome preclinical studies is a strong step towards identifying consistent biomarkers that have a high likelihood of validation by downstream studies^45^.

In conclusion, PSG and vancomycin have complex effects on the bacterial microbiome and also significant effects on the host immune response. PSG distinctly favours *C. albicans* colonization through direct effects on the bacterial microbiome. Although PSG also alters the GI immune response and fungal microbiome, these changes have a lesser influence on the level of *C. albicans* colonization of the GI tract.

## Methods

### Mouse experiments

Male C57BL/6 mice (Taconic Farms) were housed in high efficiency particulate air (HEPA) filtered cages with sterilized bedding and fed autoclaved chow and water ad libitum throughout the experiment. Prior to antibiotic treatment, mouse faecal pellets were plated onto Sabouraud agar containing chloramphenicol and streptomycin to verify that the mice were not colonized with *Candida* spp. The mice were divided into a control and two treatment groups. The following antibiotics were added to the drinking water of mice in the two treatment groups and continued for the duration of the experiment: a) vancomycin (0.5 mg/ml), and b) a combination of penicillin (1.5 mg/ml), streptomycin (2 mg/ml), and gentamicin (0.1 mg/ml) (PSG) (Figure 1). After 7 days of antibiotic treatment, 10^5^ yeast cells of *C. albicans* SC5314 were administered by gavage to some of the mice. Others were continued on antibiotic treatment alone and were not colonized. All colonized mice were quarantined to prevent cross-infection. The mouse studies were carried out in accordance with the National Institutes of Health guidelines for the ethical treatment of animals. This protocol was approved by the Institutional Animal Care and Use Committee of the Los Angeles Biomedical Research Institute at Harbor-UCLA Medical Center.

### Sample collection, DNA and RNA extraction

Experiments were initiated with 10 to 12 mice in each control and treatment group. On days 7, 9, 14 and 21, freshly obtained faecal pellets were weighed, homogenized, and then quantitatively cultured on Sabouraud agar containing chloramphenicol and streptomycin to measure the number of *C. albicans* CFUs. On days 7 and 21, 3 to 5 mice per treatment group were humanely euthanized and segments of their terminal ileum were harvested. A portion of these segments was cut lengthwise and vortexed briefly in PBS to separate the loosely adherent material from the mucosa-associated contents. Microbial DNA was isolated from these contents with the QIAamp DNA Stool Minikit (Qiagen) using the protocol from Wu et al.^46^ modified to include a bead-beating step for more efficient lysis and improved nucleic acid recovery. Autoclaved mouse chow was also processed to evaluate the contribution of ingested fungal and bacterial DNA. A separate portion of the ileum segments were immersed in RNAlater (Qiagen) immediately after collection, and RNA was isolated using the RiboPure RNA Purification Kit (Life Technologies). After preparing cDNA using the RETROscript reverse transcriptase kit (Life Technologies), mRNA expression levels of IL-17A, IL-22, IL-21, TNF-α, IFN-γ and IL-4 were measured by real-time PCR and normalized using GAPDH by the ΔΔCt method. At a minimum, samples and data were available from at least 3–5 mice for each treatment arm and for each experiment. Each experiment was repeated at least twice.

### Amplification and sequencing

Primer pairs targeting the V3–V5 region of bacterial 16S rRNA gene and fungal internal transcribed spacer (ITS) region were used to amplify the extracted microbial DNA. All amplified sequences contained 10 bp unique barcodes and 454 FLX Titanium adaptors. The primer sequences and amplification strategy are described on the accompanying website^47^. The PCR products were cleaned using the Qiagen MinElute 96 UF PCR Purification Kit and quantified using the Invitrogen Quant-iT™ High-Sensitivity DNA Assay Kit. 25 ng of amplified DNA was pooled from each sample, concentrated and cleaned up to remove any residual primers or nucleotides using the Agencourt AMPure XP Kit. The amplicons were sequenced on the 454 FLX Titanium sequencing platform.

### Taxonomic classification

Taxonomic classification of the 16S and ITS sequences was performed using YAP, a distributed bioinformatics workflow^48^, described on the accompanying website^47^. YAP integrates established software modules for 16S and ITS read processing and performs several quality filtering and trimming steps including removal of ambiguous, very short (<220 bp), chimeric and non-16S sequences using implementations of PyroNoise^49^ and *uchime*^50^ within *mothur*^51^. The filtered and trimmed sequences were efficiently clustered at 97% similarity level using CD-HIT^52^ to produce operational taxonomic units (OTU). These OTUs were taxonomically classified up to the genus level using *mothur*'s implementation of the naive Bayes classifier^53^. OTUs that remained unclassified at the genus level were assigned taxonomies using the NCBI Megablast algorithm^34^. For genera of particular interest, for example, *Candida*, the most predominant species level taxonomic labels were also identified using Megablast^34^. Predominant species were the ones that were most frequent within a particular genus, belonged to the largest OTU clusters, occurred in the largest number of mice samples and obtained the lowest E-value in Megablast.

### 16S and ITS Databases

The YAP workflow incorporates high quality databases to verify 16S and ITS origin of sequences, check for chimeras and assign taxonomies. For bacterial 16S, the SILVA database^54^ and a training dataset from the RDP project^55^ were used. For fungal ITS, a custom database was built as follows. First, a seed database of the entire fungal ribosomal region consisting of 236 diverse ribosomal sequences was compiled by running NT BLAST on an annotated ribosomal region of *Aspergillus fumigatus* Af293^56^. Next, 155,136 unique ITS sequences were retrieved from the NCBI public database, aligned to the seed database and oriented in the 5′ to 3′ direction. Clustering was performed in two iterations. The first, at 100% sequence identity, yielded 149,266 clusters and the second, at 97% sequence identity, yielded 39,548 species level clusters. Annotations were reassigned for 2,153 sequences based on clustering results. Finally, 10,226 sequences taxonomically identified as “uncultured” were queried against the NCBI taxonomy database to obtain the maximum possible taxonomic resolution.

### Data processing

After confirming that each of the two independent experimental replicates showed similar trends, sequence count data and the cytokine expression data from these two independent replications were combined. The GAPDH normalized cytokine mRNA expression levels and genera level relative abundances of sequences were log transformed to bring the numerical attributes of the dataset into the same dynamic range.

### Exploratory analyses

The 99.6% bootstrap CI of the Welch's test statistic was computed to assess significant differences in microbial diversity and cytokine expression levels between each of the treatment (vancomycin, PSG) and the control groups. Differences were deemed significant when the 99.6% CI did not include zero. The CI was computed using 10,000 bootstrap resamples of the data, was free of distributional assumptions and included a conservative correction for 6 simultaneous hypotheses corresponding to the 6 cytokines. The analyses with the bacterial microbiome was performed using 30 mice; 10 in each treatment group while those with the fungal microbiome was performed on 36 mice; 12 in each treatment group. The computation of the Welch's statistic allowed for unequal variances in the treatment groups.

### Multivariable modelling

All multivariable statistical models were estimated using Bayesian Model Averaging (BMA)^57^ with a spike-and-slab prior distribution^58^ implemented in the BoomSpikeSlab R package^33^,^59^. The spike prior is based on an assumption that a sparse set of variables can explain the response. It consists of a Bernoulli distribution that specifies whether or not a variable is selected as influential. The slab prior is a Gaussian distribution that models the effect sizes of the variables, conditional on their being chosen as influential. BMA combines information from these two priors and uses a Markov Chain Monte Carlo procedure to compute a space of 10,000 likely variable configurations that explain the response^58^. The median effect size and its associated 95% Bayesian CI for each variable was computed using the distribution of its regression coefficient across the 10,000 models. The Bayesian 95% CI was free of distributional assumptions. The initial 1,000 models were excluded as burn-in^59^. The posterior inclusion probability (PIP) for each variable is the proportion of the models that selected the variable as influential. In our findings, the median effect size and its 95% Bayesian CI and the PIP are presented as the two formal measures of statistical significance and consistency for each variable.

### BMA model specifications

Three separate sets of BMA models were estimated: a) logistic regression with antibiotic treatment (PSG or vancomycin) as response and controls as reference; linear regression with b) log(mRNA cytokine expression) as response, and c) colonization levels measured in log(CFU) as response. These models were used to evaluate a) impact of antibiotics on specific microbiota and the immune response, b) influence of specific microbiota on cytokine mRNA expression c) the role of specific microbiota and cytokine mRNA expression on the level of *C. albicans* colonization. In each model, antibiotic treatment (PSG, vancomycin), exposure to *C. albicans* (+/− *C. albicans* gavage), and time-points of sampling (Day 7, 21) were included as indicator variables. Mice in control groups served as the reference baseline. Each bacterial model was estimated from 30 mice, 10 in each treatment group (controls, vancomycin and PSG). Each fungal model was estimated from 36 mice, 12 in each treatment group. All data analyses were performed in the R language for statistical computing^60^.

### Data and code availability

Sequencing data, experimental data and associated documentation are available on the accompanying website for our project^47^. Source code for our analysis is available at GitHub^30^.

## Author Contributions

J.S. and S.G.F. interpreted the analyses and wrote the manuscript. J.S. designed and implemented the statistical analysis. N.V.S. and H.L. conducted the mouse experiments and DNA/RNA extractions. S.M. conducted the sequencing experiments. S.S. designed and implemented the bioinformatics workflow. S.G.F., W.C.N., N.V.S., S.M. and L.L. designed the study. All authors reviewed the manuscript.

## Supplementary Material

Supplementary Information Supplementary Information
