# Supplementary material for: Using Bayesian modelling to investigate factors governing antibiotic-induced Candida albicans colonization of the GI tract
Source: Sci Rep. 2015 Feb 3;5:8131. doi: 10.1038/srep08131 (PMC4314636; doi:10.1038/srep08131)
Supplement: Supplementary Information [file srep08131-s1.pdf]

Supplementary Information

Using Bayesian modelling to investigate factors governing antibiotic-induced *Candida albicans* colonization of the GI tract.

Jyoti Shankar[a*], Norma V. Solis[b], Stephanie Mounaud[a], Sebastian Szpakowski[a], Hong Liu[b], Liliana Losada[a], William C. Nierman[a], Scott G. Filler[bc*]

a. J. Craig Venter Institute, Rockville, MD, USA.
b. Los Angeles Biomedical Research Institute at Harbor-UCLA Medical Center, Torrance, CA, USA.
c. David Geffen School of Medicine at UCLA, Los Angeles, CA, USA.

*Corresponding authors

Jyoti Shankar, MBBS, PhD (Email: jyoti.shankar@gmail.com)

Scott G. Filler, MD (Email: sfiller@ucla.edu)

Model Diagnostics

We examined the following model diagnostics for the BMA ensembles explaining *C. albicans* colonization to 1) assess the explanatory power of the models, and 2) to determine whether unmeasured variables substantially influenced the level of *C. albicans* colonization.

(a) **Posterior median value of the R^2 statistic**

In Bayesian ensembles, the explanatory power of the model is estimated by the posterior median of the coefficient of determination (R^2). The R^2 statistic can also be interpreted as the fraction of variance in *C. albicans* colonization that is explained by the variables included in the model.

(b) **Posterior inclusion probability (PIP) of the intercept**

A high PIP for the intercept signals the existence of variables that were not included in the model and whose effects on *C. albicans* colonization were homogeneous across samples. Conversely, a low PIP for the intercept shows that the variables that were not included in the model exerted little influence on *C. albicans* colonization.

In our preliminary analysis, the impact of the antibiotics on bacterial diversity was most clearly significant in the faecal pellets and not as prominent in the terminal ileum (Figure 2c). This is confirmed by the high value of R^2(0.8961) in the bacterial models estimated from the faecal pellet measurements. It indicates that the bacterial microbiome, cytokines and experimental variables included in the ensembles explained nearly 90% of the variance in *C. albicans* colonization. Only around 10% of colonization variance remained unexplained.

Table S1: Model Diagnostics

	Posterior Inclusion Probability (PIP) of the model Intercept (in %)	Posterior median of the coefficient of determination (R^2)
**Bacterial Models**		
Fecal pellets	0.04	0.8961
Terminal ileum	0.40	0.5287
**Fungal Models**		
Fecal pellets	99.96	0.8374
Terminal ileum	100.00	0.8482

If the variables that were not included in the model had constant effects on *C. albicans* colonization, we would expect these effects to be represented by the intercept term. Furthermore, we would expect the intercept to have a high PIP if these variables were influential on *C. albicans* colonization. However, in the bacterial faecal pellet BMA ensemble, the PIP of the intercept was close to 0 ($PIP_{intercept} = 0.04\%$). This shows that the effects of variables not included in the ensemble were neither homogeneous nor substantial.

In the terminal ileum, the effect of antibiotics was not substantial in the presence of *C. albicans* (Figure 2c). This observation is confirmed by the lower R^2 (0.5287) seen in the bacterial terminal ileum BMA ensemble. Similar to the bacterial faecal pellet ensemble, the PIP of the intercept was less than 1% ($PIP_{intercept}$= 0.4%). This implies that the variables not included in the model did not show homogeneous effects across samples and groups and were not a substantial influence on *C. albicans* colonization.

In contrast, if we examine the diagnostics of the fungal models, we note that the PIP of the intercept is nearly 100% which indicates that the variables not included in the model

and represented by the intercept were highly influential on *C. albicans* colonization and had homogeneous effects across samples and groups. The high R^2 ($>$0.8) in the fungal models arises from the indicator variables for antibiotics and *C. albicans* gavage, each of which has a PIP of 100% (Figure 6c and 6d). Taken together with the findings from the bacterial models, which did not include any fungal variables, we can deduce that the intercept in the fungal model represents the bacterial variables.

From these model diagnostics, we can infer that the BMA ensembles explaining *C. albicans* colonization had very good explanatory power. In addition, the models estimated using the bacterial microbiome included a large fraction of the relevant variables influential on *C. albicans* colonization levels.
